# Can Co-Activation of Nrf2 and Neurotrophic Signaling Pathway Slow Alzheimer’s Disease?

**DOI:** 10.3390/ijms18061168

**Published:** 2017-05-31

**Authors:** Kelsey E. Murphy, Joshua J. Park

**Affiliations:** Department of Neurosciences, College of Medicine and Life Sciences, University of Toledo, Toledo, OH 43614, USA; kelsey.murphy@rockets.utoledo.edu

**Keywords:** Alzheimer’s disease, amyloid peptide, mitochondrial damage, oxidative stress, Nrf2, neurotrophic signaling pathway, natural products

## Abstract

Alzheimer’s disease (AD) is a multifaceted disease that is hard to treat by single-modal treatment. AD starts with amyloid peptides, mitochondrial dysfunction, and oxidative stress and later is accompanied with chronic endoplasmic reticulum (ER) stress and autophagy dysfunction, resulting in more complicated pathogenesis. Currently, few treatments can modify the complicated pathogenic progress of AD. Compared to the treatment with exogenous antioxidants, the activation of global antioxidant defense system via Nrf2 looks more promising in attenuating oxidative stress in AD brains. Accompanying the activation of the Nrf2-mediated antioxidant defense system that reduce the AD-causative factor, oxidative stress, it is also necessary to activate the neurotrophic signaling pathway that replaces damaged organelles and molecules with new ones. Thus, the dual actions to activate both the Nrf2 antioxidant system and neurotrophic signaling pathway are expected to provide a better strategy to modify AD pathogenesis. Here, we review the current understanding of AD pathogenesis and neuronal defense systems and discuss a possible way to co-activate the Nrf2 antioxidant system and neurotrophic signaling pathway with the hope of helping to find a better strategy to slow AD.

## 1. Introduction

Alzheimer’s disease (AD) is a devastating neurodegenerative disease that impairs memory, reasoning, and judgment and causes cognitive defect and behavioral changes. AD affects 35.6 million people worldwide and its incidence is expected to increase to 115 million people by 2050. In spite of the upcoming surge of AD incidence, there is still no disease-modifying treatment for AD.

Removing the AD trigger is thought to be a way to modify AD progress. However, it is still unclear what triggers AD. Moreover, there appears to be more than one trigger, which necessitates multi-modal strategy for AD treatment. Furthermore, new pathogenic factors show up in the middle of AD pathogenesis, which makes it even harder to control AD. For example, soluble amyloid beta (Aβ) peptides that are formed at early AD stage initiate multiple vicious cycles that reduce synaptic transmission, damage mitochondria, and increase oxidative stress. Heightened oxidative stress is, then, followed by chronic endoplasmic reticulum (ER) stress response and autophagy dysfunction. The congregation of all those problems makes AD more complicated and untreatable by current therapeutic approaches.

In accord with the complicated pathogenesis of AD, single-modal treatment has been ineffective in controlling AD. Failure of supplemental antioxidants in treating AD is an example of ineffective single-modal treatment. Administered exogenous antioxidants appear to be easily overwhelmed by free reactive radicals that are generated in an unregulated manner due to the loss of endogenous antioxidant system. Therefore, a more fundamental solution is required to cope with overwhelming oxidative stress. Activating nuclear factor erythroid 2 (NF-E2)-related factor 2 (Nrf2), the main switch of intracellular antioxidant defense system, may be a reasonable strategy to attenuate oxidative stress globally. That is why researchers have tried to find an effective AD treatment among flavonoids, polyphenols, and compounds that can activate Nrf2 and antioxidant response element (ARE) pathway.

To further modify AD progress, reducing oxidative stress alone may not be sufficient. Regenerative force should be accompanied for repairing damaged mitochondria and rebuilding atrophied neuronal structures. The neurotrophic signaling pathway is the major route used by neurons to regenerate their structures to regain normal synaptic transmission. Therefore, activation of the neurotrophic signaling pathway along with the Nrf2-ARE system should provide a better chance to change the course of AD pathogenesis.

Here, we will briefly review the current understanding of AD-causing factors, key neuronal defense systems, and natural products that activate the defense systems. The most recent literatures that cover each topic relatively well were used as references in our review paper. On the basis of our understanding, we discuss a possible way to modify AD progress by co-activating the Nrf2-ARE and neurotrophic signaling pathway.

## 2. AD-Causing Factors

**Figure 1:**
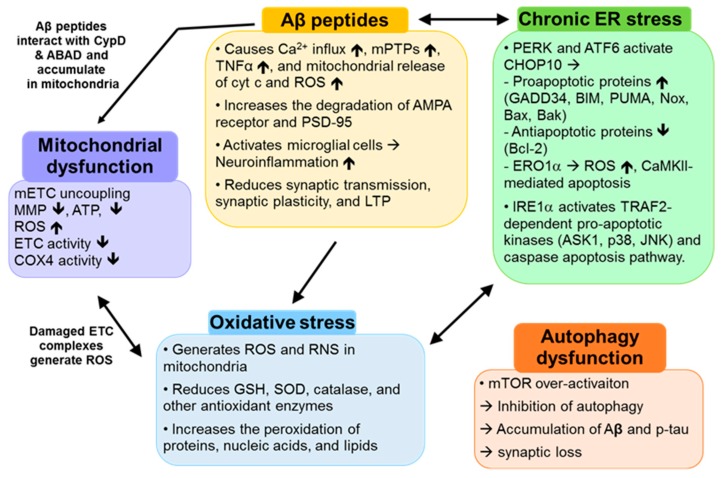
Multifaceted Alzheimer’s Disease (AD) pathogenesis. Aβ peptides increase calcium influx, mitochondrial permeability transition pore (mPTP) formation, tumor necrosis factor α (TNFα), and mitochondrial cytochrome c (cyt c) and reactive oxygen species (ROS) release, reduce α-amino-3-hydroxy-5-methyl-4-isoxazole-propionic acid (AMPA) receptor and postsynaptic density protein 95 (PSD95), and activate microglial cells that in turn induce neuroinflammation. All these negative effects lead to the gradual loss of synaptic transmission and plasticity and long-term potentiation (LTP). Aβ peptides directly attack mitochondria via its interactions with cyclophilin D (CypD) and Aβ-binding alcohol dehydrogenase (ABAD), thus causing the uncoupling of mitochondrial electron transport chain (mETC), the reduction of mitochondrial membrane potential (MMP) and adenosine tri-phosphate (ATP), and the loss of ETC enzymes including cytochrome c oxidase 4 (COX4), which result in mitochondrial dysfunction. Damaged mitochondria release ROS and reactive nitrogen species (RNS) which reduce the antioxidant enzymes and increase the peroxidation of intracellular molecules. Both chronic oxidative stress and Aβ peptides are followed by chronic endoplasmic reticulum (ER) stress. During chronic ER stress, protein kinase RNA like ER kinase (PERK) and activating transcription factor 6 (ATF6) activate C/EBP homologous protein-10 (CHOP10) that, in turn, increases pro-apoptotic proteins (growth arrest and DNA damage-inducible protein 34 [GADD34], B-cell lymphoma 2 (BCL-2) interacting mediator of cell death [BIM], p53 upregulated modulator of apoptosis [PUMA], Noxa, Bax, Bak, and ER oxidase 1α [ERO1α]) and decreases anti-apoptotic protein, Bcl-2. During chronic ER stress, inositol-requiring kinase 1α (IRE1α) activates tumor necrosis factor receptor-associated factor 2 (TRAF2)-dependent pro-apoptotic kinases, apoptosis signal-regulating kinase 1(ASK1), p38, and c-Jun N-terminal kinase (JNK) and caspase-mediated apoptosis. Chronic ER stress also contributes to oxidative stress. In AD brains, autophagy becomes dysfunctional due to the over-activation of mammalian target of rapamycin (mTOR). Inhibition of autophagy results in the accumulation of Aβ peptides and p-tau and the loss of synapses. (black arrows: cause/contribute to).

We summarized the effects of AD-causing factors and their crosstalk in Figure 1.

### 2.1. Aβ Peptides

Accumulated evidence indicates that soluble Aβ peptide oligomers rather than insoluble Aβ peptide fibrils are a major AD-causing factor [1]. Soluble Aβ peptide oligomers perturb calcium homeostasis by inducing massive calcium influx [2], which leads to the formation of mitochondrial permeability transition pores (mPTPs) in the inner mitochondrial membrane, resulting in depletion of adenosine tri-phosphate (ATP), release of cytochrome c (cyt c) and free radicals, and oxidative stress [3]. Aβ peptide oligomers also increase tumor necrosis factor α (TNF-α) and activate the pro-apoptotic kinase, c-Jun N-terminal kinase (JNK), in AD brains [4]. Moreover, Aβ peptide oligomers infuriates microglial cells that, in turn, release free radicals and inflammatory cytokines [5]. All these effects cause oxidative damage to DNA, proteins, and lipids in neurons and eventually their death [6]. In addition to the pro-apoptotic effects, soluble Aβ peptides acutely inhibit synaptic transmission. High levels of Aβ peptides decrease synapse strength by increasing the endocytosis and degradation of α-amino-3-hydroxy-5-methyl-4-isoxazole-propionic acid (AMPA) receptors [7]. Aβ peptides also cause the *N*-methyl-d-aspartate (NMDA)-mediated degradation of postsynaptic density protein 95 (PSD-95) [8]. All these negative effects of Aβ peptides lead to the reduction of synaptic plasticity and long-term potentiation (LTP) [9], the increase of long-term depression (LTD) [10], and memory deficit [11]. In addition to the above negative effects, soluble Aβ peptides exert other detrimental effects that will be reviewed in later sections.

### 2.2. Mitochondrial Dysfunction

Mitochondrial dysfunction is another major facilitator of AD pathogenesis [12]. It starts as early as 3 months of age in AD mice when intracellular Aβ peptides become detectable [13]. Mitochondrial dysfunction begins with the uncoupling of mitochondrial electron transport chain (mETC) and the depolarization of mitochondrial membrane potential (MMP), which results in the production of reactive oxygen species (ROS) and the depletion of ATP [14]. Cytochrome c oxidase (COX) in the complex IV of mETC is the most affected ETC enzyme in AD [15]. ETC complex I was also found defective in P301L tau mice, amyloid precursor protein (APP)swe/presenilin 1 (PS1)dE9 mice, Tg4510 AD mice, and AD patients [16,17,18].

Aβ peptides that directly damage mitochondria are recruited to mitochondria by the translocase of the outer membrane (TOM) machinery [19], receptor for advanced glycation end-products (RAGE) [20], and ER-mitochondria cross-contact [21]. After recruitment, Aβ peptides perturb mitochondrial function via its interaction with cyclophilin D (CypD) [22]. Similarly, the interaction of Aβ peptide with Aβ-binding alcohol dehydrogenase (ABAD) also perturbs mitochondrial function [23]. Aβ peptides accumulated inside mitochondria [24] causes the reduction in oxygen consumption and ETC activity [25]. The cortex and hippocampus are the major brain areas where Aβ peptides are accumulated inside mitochondria [26,27].

### 2.3. Oxidative Stress

During aging, endogenous antioxidant capacity is gradually reduced and then overwhelmed by oxidative stress [28]. Loss of antioxidant capacity occurs more quickly in AD brains [28]. For example, the levels of NAD(P)H, an ETC electron donor, and glutathione (GSH) were reported to be reduced more quickly in the hippocampus and cortex of 3xTg-AD mice than in wild-type mice [29]. Since oxidative stress in general is reviewed elsewhere [30,31], we will only briefly review it here.

During oxidative stress, mitochondrial ETC complexes and cytoplasmic enzymes generate ROS such as superoxide (O_2_^•−^) and hydrogen peroxide (H_2_O_2_) and reactive nitrogen species (RNS) such as nitric oxide (^•^NO), dinitrogen tetroxide (N_2_O_4_), and peroxynitrite (ONOO^−^) [32]. O_2_^•−^ is generated mainly by mitochondrial complexes I and III [33]. O_2_^•−^ is reduced to OH^•^ and OH^−^. O_2_^•−^ and ^•^NO spontaneously react with each other to generate ONOO^−^. ONOO^−^ is decomposed to generate nitrogen dioxide radical (NO^•^_2_) and ^•^NO [34]. ^•^NO was reported to impair mitochondrial respiration by inhibiting mitochondrial enzymes including COX [35]. ROS and RNS also cause the peroxidation of proteins, nucleic acids, and lipids, thus inhibiting their normal functions [36,37,38,39]. ROS produces carbonyl proteins while RNS causes protein tyrosine nitration (3-nitrotyrosine (3-NT)). High levels of protein carbonylation and 3-NT have been found in the temporal gyri, hippocampus, parietal lobes [40,41] and cerebral cortex [42] in AD patients. Increased ROS is also accompanied with the reduction in the expression and activity of GSH, superoxide dismutase (SOD), and catalase [43,44]. When ROS oxidizes DNA, it generates DNA adducts containing 8-hydroxy-2-deoxyguanine (8-OHdG) that are often found in AD brains [45].

Lipid peroxidation in AD brains occurs mainly by oxidation of polyunsaturated fatty acids in lipid membrane, which produces malondialdehyde (MDA), 4-hydroxy-2-nonenal (4HNE), acrolein, and F2-isoprostanes (F2-IsoPs). The levels of MDA, thiobarbituric acid reactive substances (TBARS), 4HNE, and HNE-modified proteins were found at high levels around neurofibrillary tangles (NFTs) and senile plaques in AD and mild cognitive impairment brains [46,47]. Similarly, acrolein [47] and F2-IsoPs [48] were found at high levels in AD brains.

### 2.4. Chronic ER Stress

Chronic oxidative stress causes the accumulation of unfolded proteins in the ER, which activates ER stress response [49]. During normal ER stress response, glucose-regulated protein 78 (GRP78/Bip) that is saturated with unfolded proteins releases three ER stress sensors, protein kinase RNA like ER kinase (PERK), inositol-requiring kinase 1α (IRE1α), and activating transcription factor 6 (ATF6) [50]. Those ER stress sensors increase the expression of ER chaperones, inhibit the entry of proteins into the ER, stop translation, and increase protein export from the ER for degradation [50]. More specifically, PERK phosphorylates the subunit of eukaryotic translation initiation factor 2α (eIF2α), thus reducing global protein synthesis to prevent further accumulation of unfolded proteins [51]. When ER stress is resolved, phospho-eIF2α (p-eIF2α) is dephosphorylated and inactivated by the phosphatase, growth arrest and DNA damage-inducible protein 34 (GADD34) [52]. Intriguingly, apart from the inhibition of global protein expression, p-eIF2α increases the expression of ATF4 [53] that induces the expression of proteins associated with redox homeostasis, energy metabolism, and protein folding during ER stress [54].

During ER stress response, IRE1α mediates the splicing of the mRNA encoding X-box binding protein 1 (XBP-1) into several XBP isoforms that activate the transcription of proteins involved in ER expansion, protein processing, folding, and exporting, and misfolded protein degradation [55,56]. IRE1α also reduces protein synthesis by inducing the degradation of mRNAs using its RNAase activity [57]. Conversely, ATF6 is transported to the Golgi complex during ER stress and cleaved by site-1 and site-2 proteases into ATF6α and ATF6β [58]. ATF6s, in turn, bind to the ER stress response element for the expression of ER chaperones and pro-survival proteins such as GRP94, protein disulfide isomerases (PDIs), XBP-1, and C/EBP homologous protein-10 (CHOP10) [59,60].

In AD brains, ER stress response appears to be chronically activated. The sustained activity of PERK and ATF6 activate CHOP10 [61,62] that, in turn, induces the expression of pro-apoptotic factors such as GADD34, B-cell lymphoma 2 (BCL-2) interacting mediator of cell death (BIM), p53 upregulated modulator of apoptosis (PUMA), and Noxa [63]. CHOP10 also down-regulates Bcl-2 and up-regulates the pro-apoptotic proteins, Bax and Bak [64]. Bax and Bak form oligomeric pores on the mitochondrial outer membrane, thus causing the release of cyt c [65,66], the loss of MMP, and the activation of caspase-9 cascade [67]. GADD34 causes the de-phosphorylation of p-eIF2α [68], which allows excessive protein synthesis and overloads ER with unfolded proteins [69]. CHOP10 also up-regulates ER oxidase 1α (ERO1α) that generates excessive ROS and, thus, depletes GSH [70]. ERO1α also activates ER Inositol-1,4,5-trisphosphate receptor on mitochondria and causes the influx of Ca^2+^ into mitochondria [71], thus increasing ROS and activating Ca^2+^/calmodulin-dependent kinase II (CaMKII)-mediated apoptosis pathway [72]. In addition, chronically activated IRE1α interacts with tumor necrosis factor receptor-associated factor 2 (TRAF2) and activates pro-apoptotic kinases including apoptosis signal-regulating kinase 1 (ASK1), p38, and JNK [73,74]. TRAF2 also causes the release of pro-caspase-12 that activates caspase-dependent apoptosis [74,75,76].

Several lines of evidence indicate that chronic ER stress response indeed occurs in AD brains. High levels of ER stress proteins such as GRP78, PERK, p-eIF2α, IRE1α, 70-kDa heat shock protein (Hsp70), PDI, ATF4, and CHOP10 were found in AD brains [77,78,79,80]. Elevated levels of p-eIF2α, ATF4, and protein kinase double-stranded RNA-dependent (PKR) were found to be associated with memory defects [81,82,83]. It was reported that excessive p-eIF2α caused cognitive defects [84], increased β-secretase 1 (BACE1), and promoted amyloidogenesis [85]. Chronic ER stress was also reported to cause tau hyper-phosphorylation in AD brains [86].

### 2.5. Autophagy Dysfunction

Autophagy is activated by the cytoplasmic accumulation of misfolded proteins [87]. Misfolded cytoplasmic proteins are engulfed into multi-membrane vesicles and delivered to lysosomes for degradation [88]. Mammalian target of rapamycin (mTOR) is a main regulator that inhibits autophagy under resting condition [87]. mTOR belongs to two different mTOR complexes, mTOR complex 1 (mTORC1) and mTORC2 [89]. AMP-activated protein kinase (AMPK) [90] and phosphoinositide 3 phosphate kinase (PI3K)/Akt [91] inhibit mTORC1 by phosphorylating mTOR, thus activating autophagy. Autophagy starts with the disassembly of the complex of Unc-51 like kinase 1 (ULK1), autophagy-related protein 13 (ATG13), and focal adhesion kinase (FAK)-family interacting protein 200 (FIP200) [92]. Released ULK1 initiates membrane nucleation by initiating its interaction with PI3KIII, Beclin-1, and ATG6 [93]. Autophagosome membrane is elongated by ubiquitin-like conjugation reactions that are mediated by E1- and E2-like conjugating ATG enzymes [94]. Then, microtubule-associated protein-1A/1B light chain 3 (LC3) is processed to LC3-I that is, in turn, lapidated into LC3-II by ATG complex [95]. LC3-II associates with lipid membrane and mediates the elongation and closure of autophagosomal membrane [96]. In addition, p62 (Sequestosome1) recruit polyubiquitinated proteins to elongating autophagosomes [97].

In AD brains, mTOR is hyperactivated, resulting in the inhibition of autophagy [98], which appears to cause the accumulation of Aβ and p-tau, synaptic loss, and cognitive decline [98]. In line with the speculation of the contribution of hyperactive TOR to AD pathogenesis, inhibition of mTOR can attenuate AD progress. Inhibition of mTOR could restore autophagy [99,100], reduce BACE1 and Aβ peptides [101] and p-tau aggregates [102], and attenuate cognitive deficits [103] in AD brains.

## 3. Key Neuronal Defense Systems

**Figure 2:**
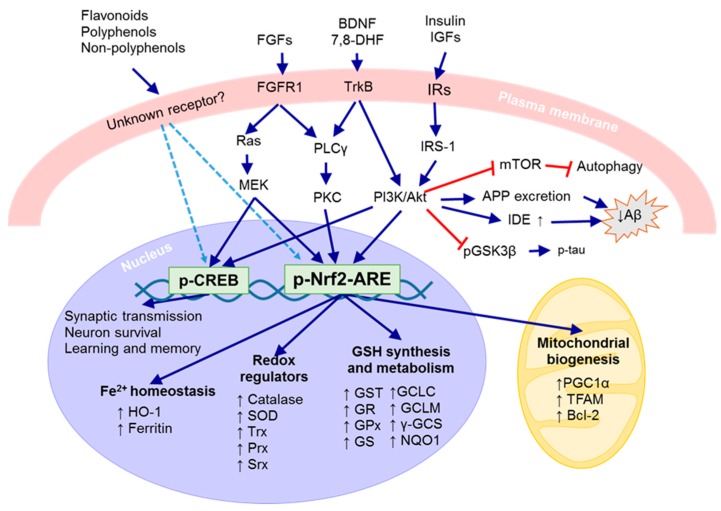
Neuronal defense systems. A group of natural products appear to activate unknown signaling pathways (dotted lines) that lead to the activation of neurotrophic (CREB) and antioxidant (Nrf2-ARE) defense systems in neurons. FGFR1 activated by fibroblast growth factors (FGFs) induces Ras-MEK and PLCγ-PKC signaling pathways that activate CREB and Nrf2. TrkB activated by brain-derived neurotrophic factor (BDNF) and 7,8-DHF induces PLCγ-PKC and PI3K-Akt signaling pathways that activate CREB and maybe Nrf2. Insulin receptors (IRs) activated by insulin or IGFs induce IRS-1-PI3K-Akt signaling pathway that activates CREB and maybe Nrf2. Activated Akt inhibits mTOR to activate autophagy, increases APP excretion and IDE expression to reduce Aβ peptides, and inhibits GSK3β to reduce p-tau. Activated CREB enhances synaptic transmission, neuron survival, and learning and memory. Activated Nrf2 binds to ARE and increases the expression of antioxidant and detoxifying enzymes involved in Fe^2+^ homeostasis, redox regulation, and GSH synthesis and metabolism. Activated Nrf2 also enhances mitochondrial biogenesis by increasing the expression of PGC1α, TFAM, Nrf1, and Bcl-2. (↑: increases; blue arrows: activate/cause; red lines: inhibit; light blue dotted arrows: may activate).

We summarized neuronal defense systems such as antioxidant defense systems and neurotrophic signaling pathways in Figure 2.

### 3.1. Antioxidant Defense System

To fight oxidative stress, neurons need to activate endogenous antioxidant defense system, especially, Nrf2, the main switch for the expression of a majority of endogenous antioxidant enzymes [104,105,106]. Under resting condition, Nrf2 is sequestered by Kelch-like ECH-associated protein 1 (Keap1) and targeted for rapid ubiquitin-mediated degradation [107]. Upon oxidative stress, Nrf2 is released from microtubule-associated Keap1 after the phosphorylation of Nrf2 and the modification (*S*-nitrosylation) of Keap1 [108]. Then, p-Nrf2 translocates into the nucleus, dimerizes with Maf, and binds to AREs in the promoters of the genes that encodes proteins involved in iron homeostasis (heme oxygenase 1 (HO-1) and Ferritin), redox regulation (catalase, peroxiredoxin (Prx), sulfiredoxin (Srx), thioredoxin (Trx), and SOD), and glutathione synthesis (glutathione *S*-transferase (GST), glutathione reductase (GR), glutathione peroxidase (GPx), glutathione cysteine ligase regulatory subunit (GCLC), glutathione cysteine ligase modulatory subunit (GCLM), and glutathione synthetase, γ-glutamyl cysteine sythetase (γ-GCS)), quinone recycling (NAD(P)H:quinoneoxidoreductase 1 (NQO1)) [109,110]. p-Nrf2 also up-regulates the expression of the genes involved in mitochondrial biogenesis such as mitochondrial transcription factors (e.g., mitochondrial transcriptional factor A (TFAM), Nrf1) [111,112].

HO has two isoforms: an inducible enzyme, HO-1, and a constitutive form, HO-2 [113]. Aβ peptide, H_2_O_2_, pro-inflammatory cytokines, and lipopolysaccharide (LPS) can induce the expression of HO-1 [114]. HO-1 showed a strong redox-controlling ability in response to ischemia, ROS, LPS [115], and Aβ peptides [116]. HOs along with nicotinamide adenine dinucleotide phosphate (NADP)H cytochrome P450 reductase catalyze the degradation of heme groups into equimolar amount of biliverdin, ferrous iron (Fe^2+^), and carbon monoxide (CO) [117]. Biliverdin is converted to the antioxidant bilirubin by biliverdin reductases (BVRs) [118]. Both biliverdin and bilirubin have strong antioxidant and anti-inflammatory effects [119]. There has been some debate about the possible contribution of HO-1 to oxidative stress because HO-1 can generate reactive iron and CO [120]. However, given that HO-1 activity is decreased at early AD stage [121], its contribution to oxidative stress is expected to be insignificant.

SOD mediates the dismutation of O_2_^•−^ to H_2_O_2_. There are two SOD isoforms, Cu/Zn-SOD (SOD1) and Mn-SOD (SOD2). SOD1 is present in the cytoplasm, lysosomes, nucleus, and inner membrane mitochondrial space [122] while SOD2 is mostly expressed in mitochondria [123]. SOD2 plays a major role in minimizing the oxidative damage of O_2_^•−^ to mitochondria by converting O_2_^•−^ to H_2_O_2_ [124]. The deficiency of SOD2 in AD mice further increased Aβ and exacerbated cognitive defect [125,126]. Conversely, overexpression of SOD2 reduced ROS production, Aβ production, memory deficit, and LTP impairment in AD mice [127,128].

Catalase in peroxisomes mediates the conversion of H_2_O_2_ to water and oxygen [129]. Trx and GSH systems detoxicate ONOO^−^ [130]. Trx system consists of Prx, Trx, and thioredoxin reductase (Txnrd) that mediate a series of disulfide exchange reactions to reduce free radicals [131]. GSH system consists of GPx family, glutaredoxin (Grx) family, and GST family [132]. GPx reduces H_2_O_2_ to water. GSH provides an electron to GPx for H_2_O_2_ reduction and then is recycled by GR and NADPH/H^+^. GSH is also generated from glutamate, cysteine, and glycine by γ-GCS, GCL (GCLC + GCLM) and glutathione synthetase (GS). Grx reduces protein disulfides (GSSG) via a disulfide exchange reaction with the expense of GSH to GSSG [132]. GSSG is then reduced back to GSH by GR. GST catalyzes the conjugation of electrophiles, reactive alkenals, xenobiotics to GSH [133].

In AD brains, Nrf2 is primarily located in the cytoplasm and much less in the nucleus [134], suggesting that Nrf2 does not actively induce the expression of antioxidant enzymes in AD brains. In line with this, some Nrf2-dependent antioxidant enzymes such as SOD1 and catalase were found reduced in human AD brains [135]. It was shown that knockout of Nrf2 in APP/presenilin 1 (PS1) mice further increased oxidative damage [136]. On the other hand, overexpression of Nrf2 enhanced neuroprotection against Aβ toxicity and recovered spatial learning in APP/PS1 mice [137]. 18 α-glycyrrhetinic acid, an Nrf2 activator, enhanced neuron survival against Aβ stress by increasing GCL and GSH [138]. Similarly, triterpenoids that activated Nrf2 attenuated oxidative stress, inflammation, and memory deficit in Tg19959 AD mice [139]. Nrf2 in different physiological and experimental conditions is summarized in Table 1.

Taken together, all these facts point to that the activation of the Nrf2-ARE antioxidant system could be the way to reduce global oxidative stress and its related pathogenesis in AD brains. As such, people have searched for exogenous compounds for the global activation of the Nrf2-ARE defense system [140,141,142,143].

### 3.2. Neurotrophic Defense System

In addition to antioxidant defense system, neurons use neurotrophic signaling pathways to survive neurodegenerative condition. cAMP response element binding protein (CREB) is a major transcription factor in neurotrophic signaling pathways [144,145]. Upon activation by phosphorylation, phospho-CREB (p-CREB) enters the nucleus and induces the expression of proteins required for neuron survival [146] and synaptic transmission [144,145]. Extracellular signal-regulated kinase (ERK) is another activator of neuron survival signaling pathway [147,148]. Upon phosphorylation, p-ERK enters the nucleus and activates transcription factors including CREB required for neuroprotection [149]. The following protein growth factors are the major activators of the neurotrophic signaling pathways for neuron survival.

#### 3.2.1. Brain-Derived Neurotrophic Factor (BDNF)

BDNF is the primary neurotrophic growth factor that enhances synaptic plasticity and memory in adult brain [150,151] by activating tropomyosin-related kinase B (TrkB) receptor [152]. Activation of TrkB leads to the activation of PI3K/Akt, phospholipase-γ (PLC-γ), ERK [153], and CREB [154]. BDNF was shown to protect adult CA1 hippocampal neurons from traumatic and ischemic brain injuries [155,156] and glutamate toxicity [157], and enhance the survival of basal forebrain cholinergic neurons [158]. However, the expression of BDNF was significantly decreased in AD patients [159,160] and AD animals [161,162]. There have been various efforts to deliver BDNF into AD brains while their efficacy has been limited by the poor blood brain barrier (BBB)-permeability and short half-life of BDNF [163].

#### 3.2.2. Insulin and Insulin-Like Growth Factor (IGF)

Insulin was shown to enhance synapse formation and neuron survival [164,165] and improve learning and memory [166] and cognitive function [167]. IGF showed similar effects to insulin [168]. Insulin, IGFs, and their receptors are expressed in neurons [169] and in the olfactory bulb, hypothalamus, cerebral cortex, cerebellum and hippocampus [168,170]. Activation of insulin receptor (IR) by either insulin or IRS-1 leads to the autophosphorylation of IR [168,170]. p-IR phosphorylates insulin receptor substrate (IRS) [170] that, in turn, activates PI3K-Akt [171]. Activated PI3K-Akt enhanced synaptic plasticity and memory consolidation [172], protected mitochondria [173], and reduced mitochondrial dysfunction and free radical production [174]. Activated PI3K-Akt also reduced Aβ peptides by increasing the expression of insulin-degrading enzyme (IDE) that degrades Aβ peptide [175,176] and the excretion of APP [177]. In addition, activated PI3K-Akt reduced p-tau production by inhibiting glutathione synthase kinase 3β (GSK3β) that generates p-tau [178]. Since insulin has a good BBB-permeability, it has been administered to AD patients via intranasal administration [179]. Insulin shows some efficacy in improving memory and cognition in early AD patients [180,181].

#### 3.2.3. Fibroblast Growth Factors (FGFs)

FGF2 was shown to activate CREB via mitogen-activated protein kinase (MAPK) and PI3K/Akt, thus enhancing neuron survival [182]. FGF2 up-regulated BDNF-TrkB-ERK-CREB signaling in retinal ganglion cells [183] and in olfactory receptor neural precursor cells [184]. Interestingly, FGFs are also able to enhance endogenous antioxidant system. FGF1 increased the expression of HO-1 and other antioxidant enzyme proteins in rat spinal cord astrocytes by activating Nrf2 [185]. FGF9 induced MAPK/ERK kinase (MEK)-ERK and PI3K-Akt signaling pathways to activate CREB and Nrf2 and increased γ-GCS and HO-1 [186,187]. Klotho, an activator of FGF23-FGF receptor signaling pathway, induced PI3K-Akt signaling pathway and increased Trx/Prx antioxidant system, thus protecting cells from glutamate-toxicity and oligomeric Aβ peptides in hippocampal neurons [188]. In spite of the beneficial effects of FGFs, the mitogenic effect of FGFs via the interaction of FGF receptor with neural cell adhesion molecule 1 (NCAM1) has been a problem for their clinical application [189]. Recently, flbroblast growth loop (FGL) peptide that activates only FGF receptor but not NCAM1 arose as a good alternative to FGFs [189].

## 4. Natural Compounds That Can Activate Nrf2 and/or Neurotrophic Signaling Pathway

In this section, we will briefly review natural compounds that were reported to activate the Nrf2 antioxidant system and/or neurotrophic signaling pathway. We do not cover all Nrf2-activating compounds in this review since many of those are covered by others [105,190]. The targets, outcomes, and research models for the preclinical studies using these natural compounds are summarized in Table 2.

### 4.1. Flavonoids

Flavonoids usually activate the Nrf2-ARE antioxidant system for their neuroprotective actions. *Pinocembrin*, an herb flavonoid, that showed neuroprotective effects in cerebral ischemic injury [191], glutamate toxicity [192], APPsw-overexpressing SH-SY5Y cells [193], and vascular dementia animal [194,195] appears to use the Nrf2-ARE system for its neuroprotective action [196]. Pinocembrain increased nuclear Nrf2 and activated the ARE-mediated expression of HO-1 and λ-GCS in SH-SY5Y cells, thus protecting the cells from 6-hydroxydopamine (6-OHDA)-induced oxidative stress [196]. *Naringenin*, a grapefruit flavonoid, that showed neuroprotective effects in Parkinson’s disease and AD models [197,198] increased nuclear Nrf2 and HO-1, GCLC, GCLM, and GSH in SH-SY5Y cells and inside mouse brain and, thus, exerted neuroprotection [199]. *Genistein*, an isoflavonoid, that showed a neuroprotective effect in global cerebral ischemia (GCI) cell models [200,201] and animal models [202] activated Nrf2-ARE signaling, increased HO-1, reduced 8-OHdG and 4HNE in hippocampal CA1 neurons, and improved learning and memory [202]. Genistein also induced the endothelial nitric oxide synthase (eNOS)-mediated *S*-nitrosylation of Keap1, thus releasing Nrf2 from Keap1 for the nuclear accumulation of Nrf2 in hippocampal CA1 neurons [202]. *Orientin*, a flavone, was shown to activate Nrf2, increase HO-1 and ARE signaling, reduce the levels of ROS, 3-NT, 4HNE, and 8-OHdG, and attenuate Aβ1-42 peptide-induced mitochondrial dysfunction and apoptotic pathway and cognitive defects in AD mice [203]. *Eriodictyol*, a Chinese herb flavonoid, was reported to activate the Nrf2-ARE system, increase HO-1, GCLC, and GCLM, and reduce ROS and apoptosis in Aβ peptide-exposed cortical neurons [204].

There are a group of flavonoids that can activate both antioxidant defense system and neurotrophic signaling pathway. *Luteolin* that exerted neuroprotection against H_2_O_2_ [205], Aβ peptide [206], and serum-starvation [207] was shown to activate both Nrf2-ARE signaling and neurotrophic signaling pathways in PC12 cells [207]. In PC12 cells, luteolin induced neurite outgrowth and increased the expression of growth-associated protein 43 (GAP-43) and HO-1 and the ARE-binding of Nrf2 in an ERK/PKC-dependent manner [207]. *Apigenin*, a plant flavone, suppressed oxidative stress in hippocampal neurons [208] and restored ERK-CREB signaling pathway in APP/PS1 AD mice [209]. Apigenin suppressed ROS elevation and reversed GSH depletion in kainic acid-treated hippocampal neurons in vitro and in the CA3 region of kainic acid-treated mice [208]. In APP/PS1 AD mice, apigenin attenuated deficits in learning and memory, reduced Aβ peptide production, increased the activity of SOD and GPx, and restored ERK/CREB/BDNF-mediated signaling pathway [209]. Lastly, 7,8-dihydroxyflavone (7,8-DHF) that showed neuroprotective effects in Fragile X mental-retardation gene (*fmr1*) knockout mice [210] and Tg2576 AD mice [211] has both neurotrophic and antioxidant effects as described by Moosavi et al. [190]. 7,8-DHF can induce TrkB dimerization and phosphorylation and activate PI3K-Akt-ERK/CREB signaling pathway, thus enhancing neuron survival in hippocampal, motor, and ganglionic neurons [190,212].

### 4.2. Non-Flavonoid Polyphenols

Compared to flavonoid polyphenols, non-flavonoid polyphenols appear to lean toward neurotrophic signaling pathway for their neuroprotective actions. Curcumin that showed neuroprotective effects against Aβ peptide [213] and in AD animal models [214] was reported to activate CREB-ERK signaling pathway in Aβ1-42-injected rats [215] and insulin signaling in 3xTg-AD mice [4]. In Aβ-injected rats, curcumin increased BDNF and p-ERK in the hippocampi and improved cognitive behavior in an ERK-dependent manner [215]. In 3xTg-AD mice that were feed with high fat diet (HFD) [4], curcumin reduced the HFD-induced activation of JNK and the inhibitory phosphorylation of IRS-1 (that allows PI3K/Akt activation) and ameliorated memory deficit [4]. Interestingly, *O*-demethylcurcumin, a curcumin chemical analog that has a similar neuroprotective effect to curcumin, not only attenuated Aβ peptide-induced caspase-dependent apoptosis but also reduced the expression of ER stress proteins such as p-PERK, p-eIF2α, p-IRE1α, XBP-1, ATF6, and CHOP in SK-N-SH cells [216]. It suggests that neurotrophic treatment may control ER stress response. Topiramate that showed neuroprotection from focal cerebral ischemia [217] and epileptic hippocampal injury [218] protected hippocampal neurons from glutamate-mediated excitotoxicity by up-regulating BDNF, p-TrkB, p-ERK, and p-CREB [219]. Compared to other non-flavonoid polysaccharides, Harpagoside, an iridoid glycoside polyphenol, can activate both antioxidant defense systems [220] and PI3K-Akt-ERK signaling system [221]. Harpagoside decreased lipid peroxidation and increased the activity of GR and SOD and the level of GSH in the cortex and hippocampus of scopolamine (muscarinic antagonist)-treated mice [220]. In other study, harpagoside treatment increased BDNF, activated ERK and TrkB-PI3K-Akt signaling pathway, and reduced memory defect in Aβ peptide-treated rats [221]. In Aβ peptide-treated cortical neurons, harpagoside decreased neurite atrophy and apoptosis in a TrkB-dependent manner [221].

### 4.3. Non-Polyphenol Compounds

Some non-polyphenol compounds also appear to use antioxidant system and neurotrophic signaling pathway for their neuroprotective actions. Taurine, a mammalian amino acid, was shown to protect neurons from glutamate cytotoxicity, maintain MMP, and reduce ROS in SH-SY5Y cells [222]. Taurine also protected mitochondria by activating Akt-CREB-peroxisome proliferator-activated receptor gamma co-activator 1-α (PGC1α) pathway [223,224,225]. In prenatally-stressed (PS) rats that had defects in learning and memory, taurine reduced mitochondrial ROS, restored MMP, COX, ATP, and SOD2, and increased Akt-CREB signaling pathway and PGC1α expression in the hippocampi [223]. Given that PGC1α is a mitochondrial activator that upregulates the transcription of proteins (e.g., TFAM) required for mitochondrial biogenesis and respiratory function [226], taurine appears to use PGC1α to restore mitochondrial function in the brains of PS rats. *R-*α-lipoic acid, a cofactor for pyruvate dehydrogenase and α-ketoglutarate dehydrogenase, was reported to enhance the syntheses of GSH and vitamin E [227] and activate both Akt/PI3K and Nrf2-ARE signaling pathways [228]. In retinal neuronal RGC-5 cells, *R*-α-lipoic acid increased the expression of HO-1 by inducing the nuclear translocation of Nrf2 in a PI3K-dependent manner [228]. *R*-α-lipoic acid also reduced ROS, 4HNE, and cell death inside animal [228]. Allicin, a garlic ingredient, was reported to reduce Aβ-induced memory deficit [229] and activated Nrf2 [230]. Allicin attenuated tau hyperphosphorylation, ROS generation, lipid peroxidation, protein carbonylation, antioxidant enzyme reduction, PERK activation, and cognitive defect in vivo [230].

## 5. Discussion

If briefly summarizing AD pathogenesis, at early stage, Aβ peptides and oxidative stress gradually overwhelms endogenous antioxidant system, thus increasing free reactive radicals. Aβ peptides and free radicals attack mitochondria in concert with one another, thus perturbing mitochondrial ETC, depleting ATP, depolarizing MMP, generating mPTPs, and releasing ROS, RNS, and lipid peroxides. All the free reactive radicals directly damage DNA, proteins and intracellular organelles, causing synaptic defects and neuron death. Following oxidative damage and mitochondrial dysfunction, ER stress response system and autophagic process that govern proteo-homeostasis are losing their normal stance. Prolonged ER stress activates apoptotic pathways by hyper-activating ER stress controllers. Inhibition of autophagy by overactivated mTOR causes the accumulation of Aβ peptides and p-tau, resulting in synaptic loss, neuron death, and cognitive decline in AD brains. All these multimodal processes make AD untreatable to current therapeutic approaches.

There has been an enormous amount of effort to find a better treatment strategy to attenuate oxidative stress and protect mitochondria. However, those efforts could not produce the positive outcome of modifying AD yet. Nonetheless, growing evidence indicates that the upregulation of global antioxidant defense system may be better for controlling oxidative stress-related pathogenesis in AD brains. Given that Nrf2 is the main switch of the expression of the majority of antioxidant enzymes and PGC1α (mitochondria enhancer), activating Nrf2 appears to be a good therapeutic strategy to control oxidative stress in AD brains. FGFs, flavonoids, *R*-α-lipoic acid, allicin, and taurine appear to be able to activate the Nrf2-ARE antioxidant defense system.

In spite of the strength of the Nrf2-ARE antioxidant system, the system alone may not be sufficient to modify AD progress. Neurotrophic signaling pathway should be also activated to regenerate damaged organelles and molecules. As such, co-activation of the Nrf2-ARE system and neurotrophic signaling pathway is expected to generate a great synergism in changing AD progress. A group of natural products, indeed, can co-activate the Nrf2-ARE antioxidant system and neurotrophic signaling pathway; those include luteolin, apigenin, 7,8-DHF, harpagoside, taurine, and *R*-α-lipoic acid. It is unclear yet whether the products activate the neurotrophic signaling pathway and the Nrf2-ARE antioxidant system separately or not. Several lines of evidence suggest that neurotrophic signaling pathway may come upstream of the Nrf2-ARE system and control the expression of antioxidant enzymes. Clarifying how neurotrophic signaling pathway talks to the Nrf2-ARE antioxidant system would help to understand the mechanism by which the natural products activate both antioxidant and neurotrophic systems.

In addition to the identification of a multimodal-effect agent, there is another must-be-addressed question regarding the mechanism of action. How can the natural products activate the Nrf2-ARE defense system and/or neurotrophic signaling pathway? Except of 7,8-DHF, none of the neuroprotective natural products have shown their interacting cellular molecules that activate the Nrf2-ARE signaling pathway and/or neurotrophic signaling pathway. Given that the majority of signaling pathways start from plasma membrane receptors, it is very likely that the natural products have their own plasma membrane receptors. It is also possible that lipid-soluble natural products penetrate the plasma membrane and interact with intracellular signaling molecules. Identification of their cellular receptors should be given a priority in order to identify their neuroprotective mechanisms and speculate their possible off-target effects.

Although chronic ER stress and autophagy dysfunction are deeply involved in AD pathogenesis, it is still unclear whether those are AD triggers or the byproducts of AD pathogenesis. Apparently, chronic oxidative stress causes the accumulation of unfolded proteins that over-capacitate ER chaperoning system, thus triggering chronic ER stress. mTOR appears to be hyper-activated by loss of its upstream regulator such as pAkt as a result of decreased neurotrophic signaling in AD brains. Normal ER stress response system may be regain-able by neurotrophic signaling pathway in a similar way to *O*-demethylcurcumin [216]. Similarly, normal autophagic activity may be resume-able by suppressing mTOR via Akt. Therefore, activation of neurotrophic signaling pathway is expected to give additional benefits like attenuating chronic ER stress and autophagy dysfunction in addition to its neuro-regenerative effect.

**Figure 3:**
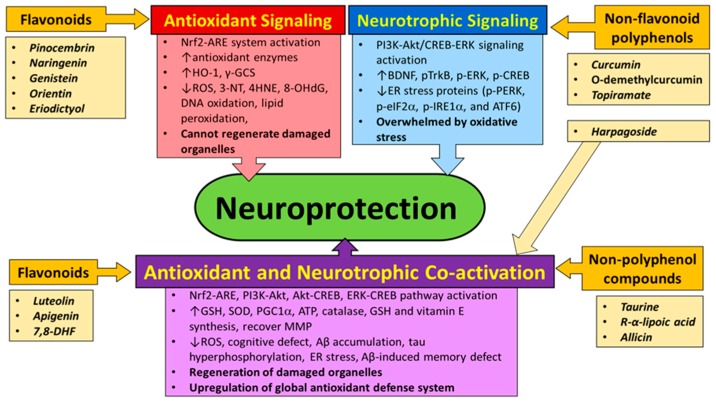
A better way to slow AD: Co-activation of the Nrf2-ARE antioxidant system and neurotrophic signaling pathway. In AD, Aβ peptides and oxidative stress increase ROS, attack mitochondrial integrity and overpower antioxidant system. This leads to generation of lipid peroxides, DNA, protein and organelle damage, synaptic defects, increased ER stress response, and ultimately neuron death. In order to mitigate oxidative stress in AD brains, global antioxidant defense system should be upregulated. The activation of the Nrf2-ARE antioxidant defense system has been seen via FGFs and certain flavonoids. However, unilateral activation of the antioxidant system is insufficient in altering AD progression. Activation of neurotrophic signaling pathway is also necessary for regeneration of damaged organelles, which has been evidenced in various non-flavonoid polyphenols. Synergistic co-activation of the Nrf2-ARE system and neurotrophic signaling pathway, however, may provide a greater ameliorating effect on AD pathogenesis. Co-activation of these pathways can be achieved by either a combination of the Nrf2-ARE activator and neurotrophic signaling activator or a multimodal activator such as luteolin, apigenin, 7,8-DHF, harpagoside, taurine, and *R*-α-lipoic acid. Either combinatory treatment or strong multimodal-effect agent will have a greater ability of ameliorating or modifying the progression of multifaceted AD by co-activating antioxidant and neurotophic signaling pathways. (↑: increase; ↓: decrease).

In this review, we try to put puzzle pieces together to help find a better strategy to slow AD. Rather than using single-modal antioxidant treatment that shows little success in slowing AD [231], co-activation of the Nrf2-ARE antioxidant system and neurotrophic signaling pathway would provide a better chance to modify the multifaceted disease, AD. Fortunately, there are some natural products that activate the Nrf2-ARE antioxidant system and/or neurotrophic signaling pathway. Although their individual efficacies in enhancing antioxidant system and neurotrophic signaling pathway vary, a combinatory treatment using both Nrf2-activating product and neurotrophic product is worthy for clinical trials. It is also worthy of finding a way to increase the potency of single natural product that can activate both antioxidant and neurotrophic systems. Based on our review, we drew a model of synergism in slowing AD pathogenesis by either the combinatory use of neurotrophic product and Nrf2-activating product or the use of a multimodal-effect agent (Figure 3). In spite of the promise, lots of works are still required for more comprehensive analysis of the neurotrophic and/or antioxidant effects of each compound prior to their clinical trials. To this end, we believe that co-activation of the Nrf2-ARE antioxidant system and neurotrophic signaling pathway may be the best chance to modify AD.

## Figures and Tables

**Table 1:** Summary of the main findings of Nrf2 in physiological and experimental conditions.

**Physiological Condition**	**Nrf2 Action**	**Reference**
Resting condition	Mediates the basal expression of antioxidant enzymes Sequestered by Keap1 Targeted for ubiquitin-mediated degradation	[104,105,106,107]
Oxidative stress	Phosphorylated Released from microtubule-associated Keap1 following the *S*-nitrosylation of Keap1 Phosphorylated and translocated into nucleus Dimerizes with Maf and binds to AREs Upregulates mitochondrial biogenesis genes	[108,109,110,111,112]
Alzheimer’s disease (human brain)	Primarily located in the cytoplasm (less in nucleus) ↓ Nrf2-mediated expression of antioxidant enzymes	[134,135]
**Experimental Condition**	**Outcome**	**Reference**
Nrf2 knockout (APP/PS1 mice)	↑ Oxidative damage	[136]
Nrf2 overexpression (APP/PS1 mice)	↑ Neuroprotection against Aβ toxicity ↑ Spatial learning and memory	[137]
Nrf2 activation via 18 α-glycyrrhetinic acid (3xTg-AD neurons)	↑ Neuron survival against Aβ stress ↑ GCL and GSH	[138]
Nrf2 activation via triterpenoids (Tg19959 AD mice)	↓ Oxidative stress, inflammation, memory deficit	[139]

(↑: increase; ↓: decrease).

**Table 2:** Natural compounds that activate Nrf2-ARE and/or neurotrophic PI3K-Akt signaling pathways.

Activator	Target	Outcome	Research Model	Reference
**Flavonoids**				
Pinocembrin	Nrf2-ARE	↑ Nuclear Nrf2, HO-1 and λ-GCS activation ↑ Protection from 6-OHDA-induced oxidative stress	SH-SY5Y cells	[196]
Naringenin	Nrf2-ARE	↑ Nuclear Nrf2 and HO-1, GCLC, GCLM, GSH	SH-SY5Y cells, C57BL/6 mouse	[199]
Genistein	Nrf2-ARE	↑ HO-1, learning and memory, ↓ 8-OHdG, 4HNE ↑ eNOS-mediated *S*-nitrosylation of Keap1 ↑ Nuclear Nrf2	GCI rat hippocampal CA1 neurons	[202]
Orientin	Nrf2-ARE	↑ HO-1 ↓ ROS, 3-NT, 4HNE, and 8-OHdG, mitochondrial dysfunction, apoptosis, cognitive defects	AD mice	[203]
Eriodictyol	Nrf2-ARE	↑ HO-1, GCLC, GCLM ↓ ROS and apoptosis	Aβ peptide- exposed cortical neurons	[204]
Luteolin *	Nrf2-ARE and neurotrophic	↑ Neurite outgrowth, GAP-43, HO-1, ARE-binding of Nrf2	PC12 cells	[207]
Apigenin *	Antioxidant and PI3K-Akt-ERK/CREB	↓ Excitotoxicity, ROS, ↑GSH ↑ SOD and GPx, learning and memory ↓ Aβ peptide production and deposition	kainic acid-treated neurons and mice APP/PS1 AD mice	[208,209]
7,8-DHF *	Antioxidant and PI3K-Akt-ERK/CREB	↑ TrkB dimerization and phosphorylation, neuron survival	hippocampal, motor, ganglionic neurons	[190,212]
**Non-Flavonoid Polyphenols**				
Curcumin	PI3K-Akt/CREB-ERK/insulin	↑ BDNF, pERK, improved cognitive behavior ↓ Active JNK, inhibitory IRS-1 phosphorylation, memory deficit	Aβ-injected rats (hippocampus) 3xTg-AD mice on HFD	[4,215]
*O*-Demethylcurcumin	Neurotrophic/ER stress response	↓ Aβ-induced caspase-dependent apoptosis ↓ ER stress protein expression (p-PERK, p-eIF2α, p-IRE1α, XBP-1, ATF6, and CHOP)	SK-N-SH cells	[216]
Topiramate	Neurotrophic	↓ Glutamate-mediated excitotoxicity ↑ BDNF, p-TrkB, p-ERK, p-CREB	hippocampal neurons	[219]
Harpagoside *	Antioxidant and PI3K-Akt-ERK	↑ GR, SOD, GSH ↓ Lipid peroxidation, memory deficit ↑ BDNF, ↓ memory defect ↓ Neurite atrophy and apoptosis	cortex and hippocampus in scopolamine- treated mice Aβ peptide- treated rats, Aβ peptide- treated cortical neurons	[220,221]
**Non-Polyphenol Compounds**				
Taurine *	Akt-CREB-PGC1α	↓ Glutamate cytotoxicity, maintain MMP, ↓ cytosolic ROS ↓ Mitochondrial ROS ↑ MMP, COX, ATP, SOD2 ↑ Hippocampal PGC1α expression, learning and memory	SH-SY5Y cells prenatally-stressed rats that showed defects in learning and memory	[222,223]
*R*-α-Lipoic acid *	Akt/PI3K and Nrf2-ARE	↑ HO-1 expression, Nrf2 translocation ↓ ROS, 4HNE, cell death	retinal neuronal RGC-5 cells	[228]
Allicin *	Nrf2-ARE and neurotrophic	↓ Aβ-induced memory deficit ↑ Nrf2, antioxidant enzymes, ↓ PERK, p-tau, ROS, lipid peroxidation, protein carbonylation, cognitive defect	AD mouse model rat brains	[229,230]

(↑: increase; ↓: decrease; * Natural compounds that can activate both Nrf2-ARE and neurotrophic signaling pathways.)

## Abbreviations

ABAD	Aβ-binding alcohol dehydrogenase
AD	Alzheimer’s disease
AMPA	α-Amino-3-hydroxy-5-methyl-4-isoxazole-propionic acid
AMPK	AMP-activated protein kinase
APP	Amyloid precursor protein
ARE	Antioxidant response element
ASK1	Apoptosis signal-regulating kinase 1
ATF6	Activating transcription factor 6
ATG	Autophagy-related protein
ATP	Adenosine triphosphate
BACE1	β-Secretase 1
BBB	Blood brain barrier
Bcl-2	B-cell lymphoma 2
BDNF	Brain-derived neurotrophic factor
BIM	Bcl-2 interacting mediator of cell death
BVRs	Biliverdin reductases
CaMKII	Calmodulin-dependent kinase II
CHOP10	C/EBP homologous protein-10
COX	Cytochrome c oxidase
CREB	cAMP response element binding protein
CypD	Cyclophilin D
cyt c	Cytochrome c
7,8-DHF	7,8-dihydroxyflavone
eIF2α	eukaryotic translation initiation factor 2α
ER	endoplasmic reticulum
ERK	extracellular signal-regulated kinase
ERO1α	ER oxidase 1α
F_2_-IsoPs	F2-isoprostanes
FAK	focal adhesion kinase
FGF	fibroblast growth factor
FGL	flbroblast growth loop
FIP200	FAK-family interacting protein 200
GADD34	Growth arrest and DNA damage-inducible protein 34
GAP-43	Growth-associated protein 43
GCI	Global cerebral ischemia
GCLM	Glutathione cysteine ligase modulatory subunit
GCLC	Glutathione cysteine ligase regulatory subunit
γ-GCS	γ-Glutamyl cysteine sythetase
GPx	Glutathione peroxidase
GR	Glutathione reductase
GRP78	Glucose-regulated protein 78
Grx	Glutaredoxin
GS	Glutathione synthetase
GSH	Glutathione
GST	Glutathione *S*-transferase
4HNE	4-Hydroxy-2-nonenal
HO-1	Heme oxygenase 1
Hsp70	70-kDa Heat shock protein
IDE	Insulin-degrading enzyme
IGF	Insulin-like growth factor
IR	Insulin receptor
IRE1α	Inositol-requiring kinase 1α
IRS	Insulin receptor substrate
JNK	c-Jun N-terminal kinase
Keap1	Kelch-like ECH-associated protein 1
LPS	Lipopolysaccharide
LTD	Long-term depression
LTP	Long-term potentiation
MAPK	Mitogen-activated protein kinase
MDA	Malondialdehyde
MEK	MAPK/ERK kinase
mETC	Mitochondrial electron transport chain
MMP	Mitochondrial membrane potential
mPTP	Mitochondrial permeability transition pore
mTOR	Mammalian target of rapamycin
mTORC	mTOR complex
NADP	Nicotinamide adenine dinucleotide phosphate
NCAM1	Neural cell adhesion molecule 1
NFTs	Neurofibrillary tangles
NMDA	*N*-Methyl-d-aspartate
NQO1	Quinone recycling (NAD(P)H:quinoneoxidoreductase 1
Nrf2	Nuclear factor erythroid 2 [NF-E2]-related factor 2,
6-OHDA	6-Hydroxydopamine
8-OHdG	8-Hydroxy-2-deoxyguanine
PDI	Protein disulfide isomerase
PERK	Protein kinase RNA like ER kinase
PGC1α	Peroxisome proliferator-activated receptor gamma co-activator 1-α
PI3K	Phosphoinositide 3 phosphate kinase
PKR	Protein kinase double-stranded RNA-dependent
PLC-γ	Phospholipase-γ
Prx	Peroxiredoxin
PS1	Presenilin 1
PSD-95	Postsynaptic density protein 95
PUMA	p53 Upregulated modulator of apoptosis
RAGE	Receptor for advanced glycation end-products
RNS	Reactive nitrogen species
ROS	Reactive oxygen species
SOD	Superoxide dismutase
Srx	Sulfiredoxin
TBARS	Thiobarbituric acid reactive substances
TFAM	Transcriptional factor A of mitochondria
TNF-α	Tumor necrosis factor α
TOM	Translocase of the outer membrane
TRAF2	Tumor necrosis factor receptor-associated factor 2
TrkB	Tropomyosin-related kinase B
Trx	Thioredoxin, Txnrd; thioredoxin reductase
ULK1	Unc-51 like kinase 1
XBP-1	X-Box binding protein 1
